# Supplementary material for: Newborns’ sensitivity to speed changes as a building block for animacy perception
Source: Sci Rep. 2021 Jan 12;11:542. doi: 10.1038/s41598-020-79451-3 (PMC7803759; doi:10.1038/s41598-020-79451-3)
Supplement: Supplementary file 1 — Supplementary Information [file 41598_2020_79451_MOESM1_ESM.pdf]

Supplementary information

"Newborns' sensitivity to speed changes as a building block for animacy perception".

Elisa Di Giorgio, Marco Lunghi, Giorgio Vallortigara, & Francesca Simion

Additional statistical analyses

We conducted additional analyses on the total number of orienting responses that indexed attention-getting mechanisms [1]. Here we reported the results for each experimental condition.

Experiment 1:

Increased speed stimulus (*M* = 17.7, *SD* = 5.7) *vs.* constant-speed stimulus (*M* = 17.1, *SD* = 6.9), t_{11} = 0.30, *p* = 0.77.

Experiment 2:

Decreased-speed stimulus (*M* = 13.4, *SD* = 4.2) *vs.* constant-speed stimulus (*M* = 13.8, *SD* = 5.3), t_{10} = 0.33, *p* = 0.75.

Experiment 3:

Stimulus that increased and the decreased its speed (*M* = 15.7, *SD* = 6.8) *vs.* constant-speed stimulus (*M* = 16.3 *SD* = 5.3),

t_{12} = 0.44, *p* = 0.67.

Experiment 4:

Stimulus that decreased and then increased its speed (*M* = 13.3, *SD* = 4.9) *vs.* constant-speed stimulus (*M* = 13.8, *SD* = 4.6),

t_{11} = 0.26, *p* = 0.80.

References

1. Cohen L.B. Attention-getting and attention-holding processes of infant visual preferences. *Child Dev*. **43**, 869–879, DOI: 10.2307/1127638 (1972).
